# Alterations in ECM signature underscore multiple sub-phenotypes of intervertebral disc degeneration

**DOI:** 10.1016/j.mbplus.2020.100036

**Published:** 2020-04-21

**Authors:** Takashi Ohnishi, Emanuel J. Novais, Makarand V. Risbud

**Affiliations:** a Department of Orthopaedic Surgery, Sidney Kimmel Medical College, Thomas Jefferson University, Philadelphia, PA, USA; b Department of Orthopaedic Surgery, Faculty of Medicine and Graduate School of Medicine, Hokkaido University, Sapporo, Japan; c Life and Health Sciences Research Institute (ICVS), School of Medicine, University of Minho, Braga, Portugal; d ICVS/3B's – PT Government Associate Laboratory, Braga, Portugal

**Keywords:** Intervertebral disc, Nucleus pulposus, Annulus fibrosus, Extracellular matrix, Disc degeneration, Fibrosis, Sub-phenotypes

## Abstract

The intervertebral disc is a specialized connective tissue critical for absorption of mechanical loads and providing flexibility to the spinal column. The disc ECM is complex and plays a vital role in imparting tissue its biomechanical function. The central NP is primarily composed of large aggregating proteoglycans (PGs) while surrounding AF is composed of fibrillar collagens, I and II. Aggrecan and versican in particular, due to their high concentration of sulfated GAG chains form large aggregates with hyaluronic acid (HA) and provide water binding capacity to the disc. Degradation of aggrecan core protein due to aggrecanase and MMP activity, SNPs that affect number of chondroitin sulfate (CS) substitutions and alteration in enzymes critical in synthesis of CS chains can impair the aggrecan functionality. Similarly, levels of many matrix and matrix-related molecules e.g. Col2, Col9, HAS2, ccn2 are dysregulated during disc degeneration and genetic animal models have helped establish causative link between their expression and disc health. In the degenerating and herniated discs, increased levels of inflammatory cytokines such as TNF-α, IL-1β and IL-6 are shown to promote matrix degradation through regulating expression and activity of critical proteases and stimulate immune cell activation. Recent studies of different mouse strains have better elucidated the broader impact of spontaneous degeneration on disc matrix homeostasis. SM/J mice showed an increased cell apoptosis, loss of cell phenotype, and cleavage of aggrecan during early stages followed by tissue fibrosis evident by enrichment of several collagens, SLRPs and fibronectin. In summary, while disc degeneration encompasses wide spectrum of degenerative phenotypes extensive matrix degradation and remodeling underscores all of them.

## Highlights

• The intervertebral disc absorbs loads and provides flexibility to the spine.
• The ECM is complex and vital for imparting tissue its biomechanical function.
• Numerous types of proteoglycans and collagens designate the quality of the disc.
• Many matrix and matrix-related molecules are dysregulated during disc degeneration.
• Matrix degradation and remodeling underscores wide spectrum of phenotype.

## Introduction

**Fig. 1:**
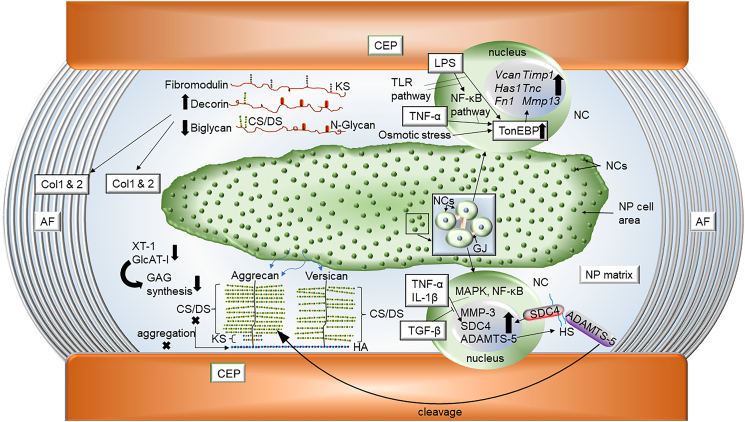
A scheme of important signaling pathways and matrix molecules that play roles in intervertebral disc health and disease. Notochordal cells (NCs) populate in the center of the nucleus pulposus (NP) and communicate with each other by many gap junctions (GJs). The annulus fibrosus (AF) and cartilaginous endplate (CEP) encapsulate the NP.

Intervertebral disc degeneration is one of the main contributors to chronic low back, and neck pain, among leading causes of disability in the United States [1,2]. The intervertebral disc and the adjoining vertebral bodies forms a polyaxial diarthrodial joint which provides flexibility and range of motion to the spinal column and accommodates the compressive biomechanical forces applied to the spine [3]. The disc is comprised of 3 distinct tissues: the nucleus pulposus (NP) – a proteoglycan rich, gelatinous tissue that forms the core of the disc and is sparsely populated with cells derived from the notochord; ligamentous annulus fibrous (AF) - that surrounds the NP and is primarily composed by fibrillar collagens; and the hyaline cartilaginous endplates (CEP), bordering the NP and AF on cranial and caudal surfaces (see Fig. 1). The interaction between these 3 compartments provide the disc its ability to absorb loads. The applied loads are resisted by the osmotic swelling pressure in the NP generated as a result of high concentration of negatively charged glycosaminoglycans that substitute large aggregating proteoglycans, aggrecan and versican and the hoop stresses in the AF that contain them [4]. Noteworthy, due to the anatomical structure of the disc, blood vessels and neurons are only present in the superficial regions of CEP and AF, but do not reach the inner AF and NP compartments. Consequently, NP cells experience hypoxia and rely on HIF-1 [5] signaling to adapt to this hypoxic and nutritionally challenging microenvironment [[6], [7], [8]]. Another defining feature of the intervertebral disc niche is its extracellular matrix with each tissue compartment having a unique composition. Accordingly, ECM maintenance is critical for intervertebral disc homeostasis and several studies have documented alteration in ECM profiles in different disc degeneration models [[9], [10], [11]]. Importantly, polymorphisms in ECM genes including ACAN, COL1, COL 2, COL9, COL11, FN, HAPLN1, THBS, CILP, ASPN; and ECM related genes: GDF5, MMP1, MMP2, MMP3, and TIMP have been correlated with human disc degeneration [12,13]. This mini review will detail the current knowledge of ECM complexity in disc homeostasis and pathology.

## Complexity of ECM in the intervertebral disc

ECM composition and structure governs intervertebral disc function. The NP is enriched in different proteoglycans; aggrecan (ACAN) is the most abundant followed by versican which is diffusely distributed in the NP and present at higher concentrations than what is found in articular cartilage [14]. Importantly, these large aggregating chondroitin sulfate proteoglycans (CSPG) confer negative charge to the NP tissue resulting in attracting water molecules [15]. As a consequence, proteoglycans are responsible for maintaining tissue hydration and viscoelasticity of healthy discs, critical in absorbance of mechanical loads. In fact, decrease levels of ACAN and other CSPGs were strongly correlated with disc aging and degeneration [16]. Furthermore, replacement of versican with a complex collagenous ECM in the NP was suggested as a hallmark of disc fibrosis and degeneration process [17]. NP cells also express other PGs such as perlecan, syndecans, glypicans, and various small leucine-rich repeat proteoglycans (SLRPs) including decorin, biglycan, fibromodulin, chondroadherin and asporin and their dysregulation can compromise disc function [14,18]. Another proteoglycan, PRG4/lubricin enriched in superficial zone of articular cartilage and meniscus is found abundantly in the disc tissues, and especially in the NP [19] however its function is not clearly understood. Dysregulation of other matrix constituents such as fibronectin (FN) [20], hyaluronan and proteoglycan link protein (HAPLN1) [21], thrombospondin (THBS) [22] and cartilage intermediate layer protein (CILP) [23] were shown to disturb disc homeostasis. While relative to proteoglycans, collagens constitute a minor component of NP tissue, importance of collagen II in the disc development should be mentioned. In *Col2a1*-null mice, the notochord fails to disintegrate within developing vertebral bodies and persists as a rod-like structure, resulting in lack of NP formation [24]. Transgenic Del1 mice harboring a 150-bp deletion in *Col2a1* showed a similar notochord phenotype with *Col2a1*-null mice along with abnormal vertebral bodies. [25]. Additionally, substitution of arginine to cysteine at position 519 in *Col2a1* causes primary osteoarthritis and osteochondrodysplasia in mice resembling phenotype in human patients carrying similar mutation [26]. There is also a report that *COL2A1* rs2276454 polymorphism is associated with an increased risk, and *COL2A1* rs1793953 might be a protective factor of developing disc degeneration in a Chinese Han population [13], however these SNPs are not validated in other ethnic populations. Consequently, Collagen II is not considered a genetic risk factor for disc degeneration which seems paradoxical and warrants further investigation. Nevertheless, these studies emphasize the importance of Col II in disc development and later in life. Altered expression of other minor collagens including collagen VI [10], collagen IX (COL9) [27], collagen X [9], collagen XI [28], collagen XII [10], collagen XV [10], and collagen XVIII [10] found in the disc also affect NP and AF structure and accelerate disc degeneration. Laminin is another constituent of the NP matrix. Laminin γ1 chain and its receptor integrin 6 subunit was localized predominantly in immature NP of pig compared to adjacent AF [29]. Whereas Laminin α5 chain, laminin receptors such as integrin α3, α6, β4 subunit and CD239 and related binding proteins (CD151) were also highly expressed in NP cells [29].

The AF is composed primarily by fibrillar collagens, namely collagen I and II, organized in concentric lamellae, which offer resistance to NP swelling [30]. However, it is important to recognize that within the AF compartment, there are zonal differences in ECM composition based on the mechanical stresses experienced by the tissue. While inner AF has a similar composition to the NP, rich in PGs and collagen type II fibers to handle higher compressive forces, outer AF region is enriched in collagen I fibers, providing resistance and tensile strength [31]. In addition to collagens, the AF matrix also contains elastin and fibrillin-1 rich microfibrillar network showing different region-specific content and arrangement [32,33]. While, elastin content was similar in outer AF and inner AF in healthy discs, it increased with degeneration and age, and the highest levels were seen in degenerated inner AF [32]. Elastin was aligned in parallel with collagen bundles in the outer AF, whereas, it was dense and often crisscrossed or cross-bridged with adjacent lamellae in the inner AF [32,34]. As for fibrillin-1 rich microfibrils, their network was completely different from region to region in nondegenerate discs [33].

It is important to recognize however that the disc matrix composition is slightly different between embryonic and adult life. During embryonic development and early post-natal life, notochordal cells that constitute NP tissue synthesize collagens 1, 2, 3, 9 and express high levels of HA and CD44 not enriched or actively synthesized in the adult NP tissue [[35], [36], [37]]. Interestingly, a *Col2a1* splice variant 2A and not 2B is predominantly expressed in rabbit embryonic disc [35] an observation further illustrated by Melrose et al. in their studies of ovine disc matrix [38]. Sandell and coworkers have reported collagen IIA expression in notochord of 42-day human embryos and in the inner AF of 54-day embryos; these authors also noted that the IIA expression persisted in 52-week embryos in central disc areas whereas collagen IIB was localized in vertebral tissue adjacent to the disc [36,37]. Melrose et al. found the highest levels of decorin and versican in the AF with equal abundance of biglycan in all zones of the fetal disc [38]. Predominance of biglycan in the fetal disc and decorin in mature disc tissues were noted along significantly lower levels of versican in adult disc specimens [38]. Additionally, the composition of the disc slightly varies with its location within the spine and with aging e.g. lower levels of PGs were evident in discs at the juncture of a mobile and less mobile spinal motion segment [39].

## ECM remodeling mechanisms in the intervertebral disc

An important consideration in maintaining disc homeostasis is the tight regulation of ECM quality control and turn-over. ECM turnover is largely controlled by various proteases and their activity modulators. Those factors include metalloproteases (MMP2 [40], MMP3 [41], MMP7 [42] and MMP13 [43]), disintegrin and metalloproteinase with thrombospondin motifs (ADAMTS4 and ADAMTS5) [44], other proteases (Htra1) [45] and tissue inhibitor of metalloproteinases (TIMPs) [12]. Likewise, inflammation related proteins, such as interleukins (IL-1and IL-6) [[46], [47], [48]], tumor necrosis factor (TNF) family [49,50] and cyclooxygenase-2 (COX2) [51] are involved in this process. Dysregulation of any of these molecules is likely to affect the remodeling and composition of disc ECM resulting in compromised mechanical properties and accelerated disease progression [52].

Furthermore, it is important to recognize that for the optimal disc function the PG concentration and charge ratio should remain as high as possible [14]. PGs should possess maximal substitution of its sulfated GAG chains, and as for aggrecan, to form large aggregates with HA [14]. Fewer and shortened CS chains can impair the ability of aggrecan to aggregate with HA, and cause further fragmentation of the PG, leading to the disc degeneration [14]. It is also noteworthy that expression of enzymes involved in GAG biosynthesis is decreased with degeneration and age, as shown by significantly decreased xylosytransferase-1 levels in aged bovine discs [14] and reduced levels of glucuronosyltransferase-I in degenerated human and aged bovine discs [14]. Elevated levels of syndecan-4 (SDC4), a small, cell surface heparan sulphate proteoglycan have been implicated in disc matrix catabolism [18,53]. In the degenerating NP, the increase in inflammatory cytokines TNF-α and IL-1β regulates SDC4 expression via MAPK and NF-κB signaling pathways [18]. SDC4 is shown selectively interacts with ADAMTS-5 and increase it efficiency of aggrecan cleavage [18,54]. SDC4 is also important in modulating TNF-dependent MMP-3 expression [18,55] (see Fig. 1).

**Fig. 2:**
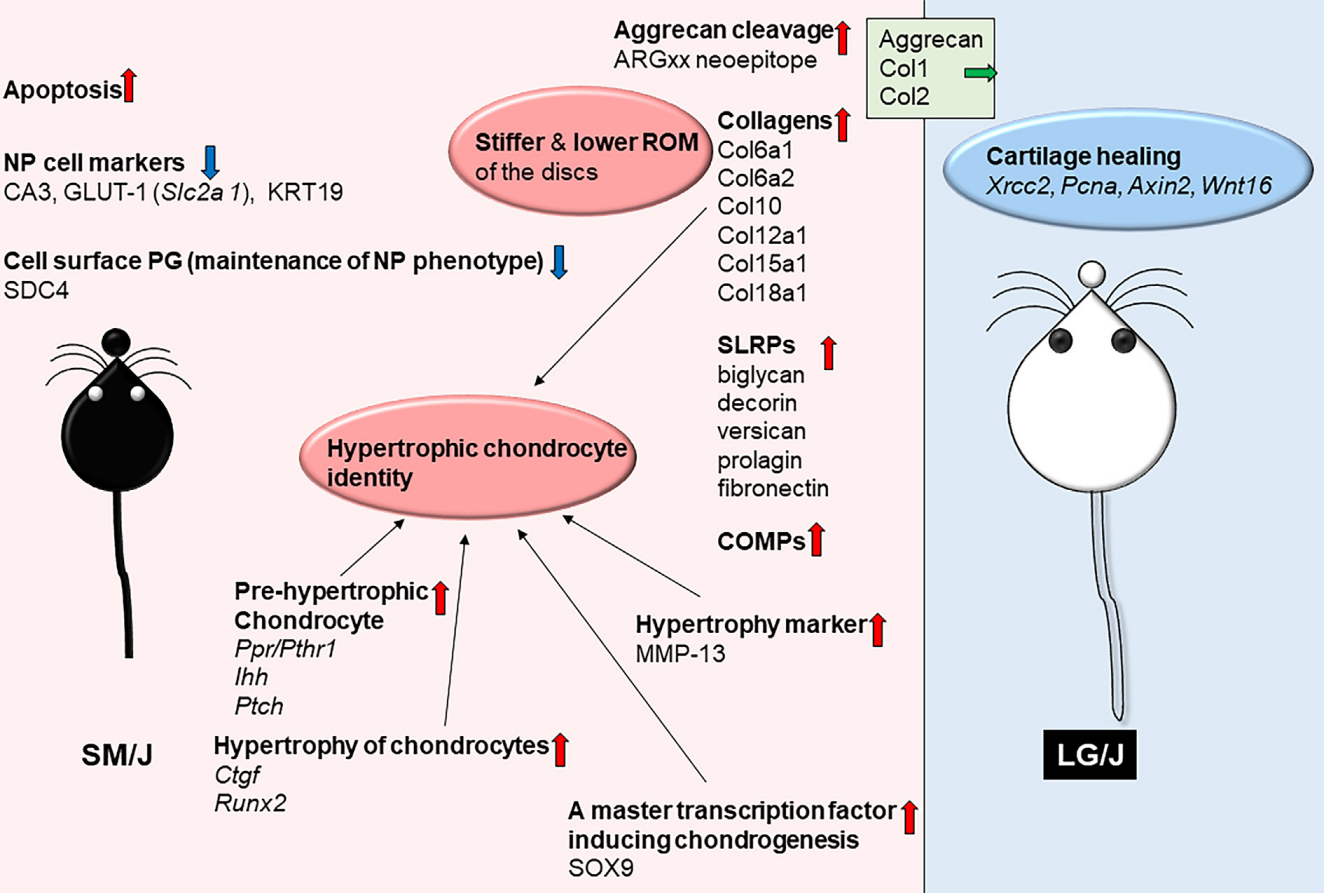
Phenotypic differences observed between SM/J and LG/J mice, mainly focusing on molecular regulation and composition of the intervertebral disc (Ref #9, 10). SM/J mice evidence spontaneous early onset disc degeneration characterized by extensive cellular and matrix changes.

**Fig. 3:**
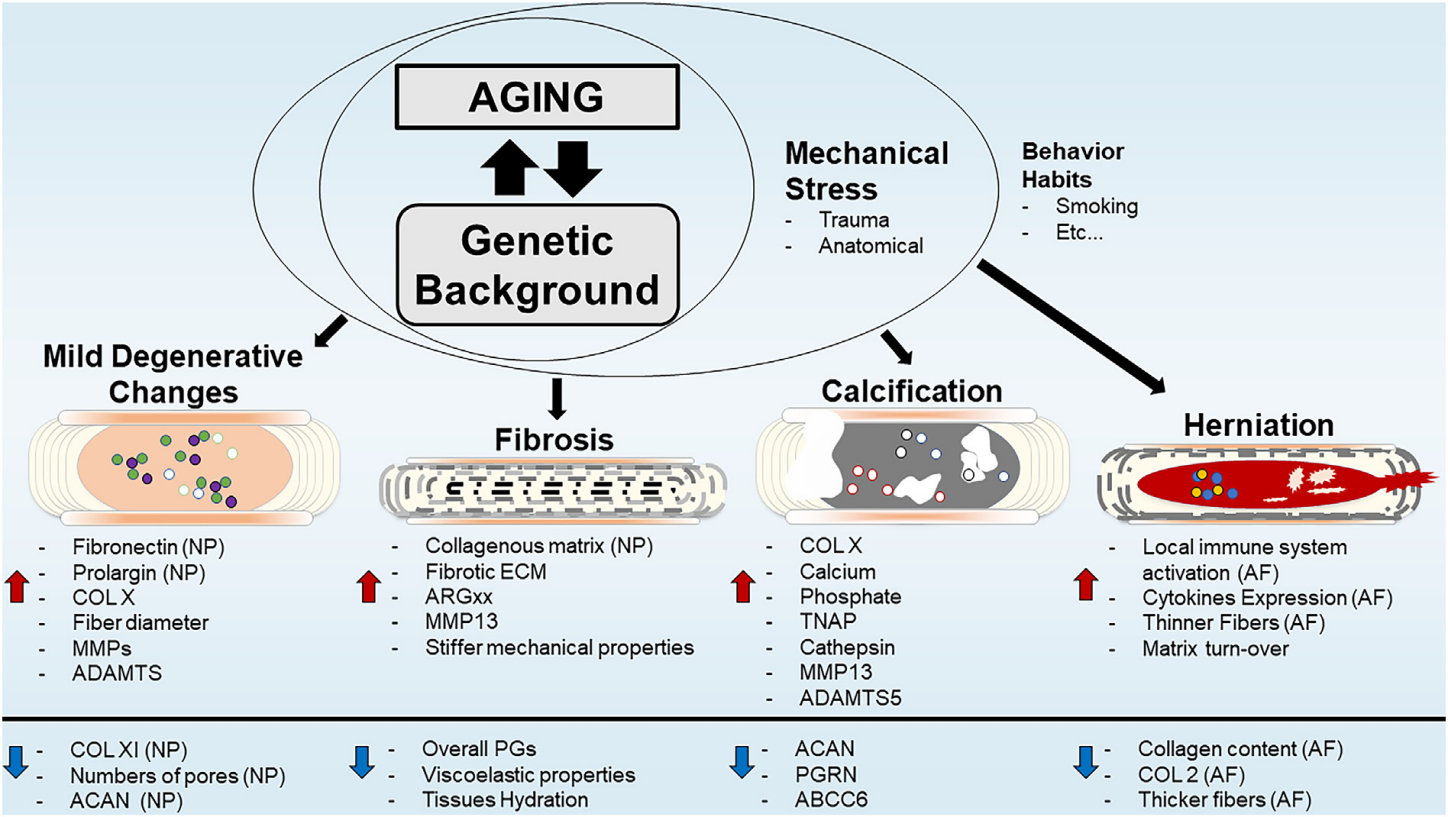
Predominant disc degeneration phenotypes and their ECM signatures. Each degenerative sub-type is characterized by a unique extracellular matrix composition and remodeling signature. Red (increase) and blue (down) arrows represent directionality of the change respectively. Abbreviations: collagen (COL); proteoglycans (PGs); tissue non-specific alkaline phosphatase (TNAP); metalloproteases (MMP); A Disintegrin and Metalloproteinase with Thrombospondin motifs (ADAMTS); aggrecan (ACAN); progranulin (PGRN); ATP-binding cassette sub-family C member 6 (ABCC6); aggrecanase (ADAMTS-1, -4 & -5)-generated N-terminal neoepitope ARG after cleavage (ARGxx); Nucleus pulposus (NP); Annulus fibrosis (AF).

An overview of various animal models clearly illustrates the importance of maintaining healthy disc matrix. Chondrodystrophic dogs lose their notochordal cells after birth and are prone to spontaneous disc degeneration [39]. This degeneration as in humans, is characterized by decreased PG content, diminished aggregation levels of PG with HA, increased keratan sulphate content, increased serine proteinase activity, and decreased serine proteinase inhibitory protein levels [39]. Likewise, biochemical changes in aging sheep and rabbits are similar to humans with respect to altered metabolism and PG synthesis [39]. More recently studies utilizing different inbred strains of mice have better elucidated the broader impact of degeneration on disc matrix. SM/J and LG/J mice exhibit contrasting phenotypes in terms of disc health [9,10]. SM/J mice exhibited an early onset spontaneous disc degeneration underscored by loss of vacuolated NP cells and their replacement with sparsely distributed chondrocyte-like cells within condensed and fibrotic matrix. This was accompanied by the loss of the AF lamellar structure, loss of AF-NP tissue boundary [9,10]. NP tissue of SM/J mice also evidenced increased apoptosis, loss of NP cell phenotypic markers, increased of ARGxx, an aggrecan degradation and collagen X abundance [9]. The analysis of the NP matrisome of SM/J mice further showed enrichment of Col6a1, Col6a2, Col12a1, Col15a1, Col18a1, versican, fibronectin, and COMP as well as biglycan and decorin, small SLRPs associated with collagen fibril formation [10]. SM/J mice were predisposed to acquire hypertrophic chondrocyte identity (see Fig. 2). Importantly, the matrix changes were accompanied by the increased stiffness and lower range of spinal motion in SM/J mice [9] (see Fig. 3).

## Matrix changes during intervertebral disc aging

Despite the well-described multifactorial etiology of disc degeneration, natural aging is still one of the major contributors to disc degeneration [9,10]. This becomes more important as advances in medicine, nutrition and public health are contributing to an increased lifespan, and therefore, to an increase in age-dependent diseases [56]. In a study including over one thousand individuals, 40% presented with disc degeneration at 30 years old, increasing progressively to over 90% by 55 years old [57]. Furthermore, it was reported that over the course of their lifespan, both humans and animal models experience decreased levels and quality of extracellular matrix proteins and biomechanical properties, as well as an increase in inflammatory cytokine expression, catabolic processes, cell death and consequently increase in degeneration [49,56]. Eyre and Muir have reported the relative amounts of collagens I and II in human thoracolumbar and lumbar discs at various ages [58]. Collagen II constituted about 50–65% of total collagen of the AF and was higher in a teen disc than that of an old disc [58]. Interestingly, the proportions of collagens I and II in the AF did not significantly differ in samples ranging from 5 years to 66 years old [58]. In contrast to AF, collagen II was more than 85% of total collagen in NP tissues regardless of age [58]. Recently, Caldeira et al. have shown an increase in collagen fiber diameter, and intersection of fibers in the NP of elder population. In the same study, after performing a matrisome analysis comparing young and old NP tissues, the authors showed that levels of fibronectin (FN) and prolargin were increased, along with decrease in collagen XI [59]. Interestingly, these results support other studies correlating FN increase with decrease in aggrecan and increase in MMP levels, promoting cartilage degradation [20]. Not surprisingly, decreased levels of aggrecan were also associated with increase of ADAMTS and MMPs activity levels during NP aging [43]. Senescence has also been reported to increase in the intervertebral disc during aging [43,47]. Senescent cells are characterized by a unique profile of catabolic and pro-inflammatory secretion, resulting in changes in disc ECM [60,61]. Recently, using disc specific conditional knockout p16INK4a mice Novais et al. showed that COL1, COL2 and CS levels were decreased, followed by an increase of COL10 in the NP [47]. Patil et al. supported this idea and showed that removal of senescent cells from aged disc leads to decrease in MMP catabolic activity, with improvement in disc health [43]. Osmotic response factors NFAT5/TonEBP [11,46] and actin regulator Arp2/3 [62] were also shown to play an important role in organizing and maintaining ECM during aging [62]. In TonEBP hypomorphic mice there was global downregulation in disc matrix genes and a noteworthy increase in COL10, an important marker of hypertrophic chondrocytes [11]. These studies along with studies of disc cell senescence, suggests a possibility that ECM shift over aging is likely driven by cell transformation. Similarly, ECM turn-over promoted by inflammatory/catabolic signaling is widely accepted. In fact, disc aging is often correlated with changes in IL-1, IL-6, MCP-1, MMPs, ADAMTS and TGF-β signaling, along with alteration in ECM composition [47]. Surprisingly, however, a recent study using IL-1α/β double KO mouse, showed that in absent of IL-1, there was a decrease in the protein levels of COL2 and COMP during aging [48]. This data suggests that cytokine levels in the disc can play a catabolic and/or anabolic role depending on the concentration levels and/or stage of the disease (healthy vs degenerated). Underscoring the importance of cytokine concentration levels and phenotypic outcome of the disc, two recent studies reported an increase in NP cellularity with and without increased incidence of NP herniation in hTNF-Tg mice [63,64]. These results suggest that during aging there is a shift in cell phenotype, promoting changes in ECM composition and remodeling, which might explain the increased incidence of disc degeneration and disease in the elder population.

## Predominant disc degeneration phenotypes

One important aspect of human disc degeneration is the wide spectrum of degenerative phenotypes. Histological analyses of human degenerated discs have shown: disc fibrosis with loss of cells, annular clefts, neovascularization, and sclerosis of the subchondral bone; ectopic calcification of the cartilaginous endplate and/or the disc itself; and herniated discs with increased senescent cells and matrix metalloproteinases expression [57,65,66]. While the contribution of genetic predisposition, tissues microenvironment, or altered mechanical environment underlying these pathological phenotypes is still unknown, ECM seems to present a unique profile in each subtype of disc disease. We describe below cellular and ECM changes seen in three such major phenotypes (see Fig. 3).

### Disc degeneration with fibrosis

Loss of tissue viscoelastic properties is one of the major hallmarks of disc degeneration. In fact, the gold standard classification of disc degeneration in clinic was proposed by Pfirrmann in 2001 [67]. This classification system of degeneration is based on signal intensity detected by T2-weighted MRI , that indirectly capture the ability of the NP to bind water. Consequently, the diagnosis of disc degeneration primarily reports the shift of an ECM rich in PGs towards a more fibrotic and stiffer ECM that is rich in collagens [57,68]. There are several contributors that can promote this turn-over of matrix. Aberrant mechanical loading is thought to play a role on stimulating disc fibrosis tissues, since in humans the prevalence of disc degeneration is higher at lumbar L5/S1 and cervical C5/C6 than the others levels [68]. Interestingly, studies have also shown that anatomical predisposition to disc degeneration is present in other species [69]. Moreover, the combination of twin cohort studies [70] and mouse stains comparisons [9,10,69] indicate that genetic background might be essential to drive disease progression towards a specific subtype of degeneration. In fact, SM/J mice, a novel model of spontaneous disc degeneration shows fewer NP cells, associated with an increase in hypertrophic chondrocyte-like phenotype [9]. This cell signature is known by secretion of preferentially collagenous ECM [71], which might explain the increased fibrotic matrix and stiffness of degenerated discs in SM/J mice [9,10]. Similarly, histological and proteomic analyses of human discs have further supported relationship among increased stiffness associated with a fibrotic environment, increased levels of collagenous matrix and number of hypertrophic like cells in degenerated disc [[72], [73], [74]].

### Degeneration with calcification

Another common subtype of disc degeneration, mainly found in elder people, is intervertebral disc calcification [75]. Indeed, histological analysis of human cadaveric samples showed that both NP and AF compartments can be affected, as well as increase in COLX, calcium, inorganic phosphate and local alkaline phosphatase activity [66]. Interestingly, deficiency of progranulin (PGRN), a pleiotropic growth factor, has been shown to result in elevation in TRAP, Cathepsin, COLX, MMP13 and ADAMTS5 levels [76]. In this case, ACAN content was also decreased, but this time in the context of intervertebral disc calcification. Importantly, studies affecting inorganic phosphate metabolism have also showed progressive ectopic mineralization of the AF and adjacent ligaments, which eventually lead to NP collapse at 1 year [77]. Although, mineralization process of the disc remains unknown, cell death, matrix remodeling, increase in local calcium and phosphate, tissue- nonspecific alkaline phosphatase (TNAP) activity and cell transformation seen in degenerating discs have been shown to contribute to mineralization [78,79]. While most models of disc degeneration present a fibrotic phenotype, LG/J mice showed an increased prevalence of intervertebral disc calcification in caudal spine during aging [80] as well as a higher predisposition of synovial and meniscus calcifications following knee trauma [81]. Importantly, LG/J disc calcification features resemble the intervertebral disc calcification seen with higher prevalence at lower thoracic levels of elder human patients at both histological and transcriptomic levels [66,75,80]. All together, these results suggest that intervertebral disc calcification phenotype underscores the combination of anatomic location, genetic predisposition and stress factor such as aging or trauma.

### Disc herniation

In humans, disc herniation is an important clinical finding associated with radicular pain, neurological changes and overall matrix catabolism processes [82]. Degeneration of the AF with subsequent changes in its structure predisposes patients to develop intervertebral disc herniation. This can result in neural compression and subsequent radicular pain [57]. Several studies have shown an important correlation between ECM composition and herniation of the disc. In fact, TonEBP deficient mice which shows a clear decrease in collagen content in the AF, presents an increased incidence of lumbar disc herniation [11]. Surprisingly, TgTNF mice, which also showed spontaneous disc herniation in caudal spine, did not present any significant changes in the ECM but only a noticeable local increase in immune system activity and cytokine expression [63,64]. Supporting the hypothesis of anatomical/mechanical loading as a predisposition factor to disc herniation, bipedal mice showed a significantly increased incidence of disc herniation [83]. Moreover, destabilization of the spine by resection of spinous processes, supraspinous and interspinous ligament, with paravertebral muscle detachment, altered AF ECM organization promoting disc herniation as well [84]. In addition, trauma induced disc herniation, usually by annular puncture, have clearly shown the importance of annulus fibrosus integrity to the pathology and have helped to further explore the progressive local response after NP herniation: inflammatory cascade, immune responses and matrix remodeling [[85], [86], [87]]. Overall, disc ECM profile and integrity seem to be influenced by the conjugation of local inflammation, mechanical stress/loading and genetic predisposition, contributing to loss of matrix integrity and consequent NP herniation.

## Conclusion

The biomechanical function of the intervertebral disc is critically dependent on a healthy ECM and it plays an essential role in cellular homeostasis by promoting signaling and survival [4,11,18,62]. In the intervertebral disc, ECM profile varies depending on anatomical position of the motion segment as well as mechanical properties of each region of the intervertebral disc. In fact, disc ECM is composed by a huge diversity of PGs, different collagens and a unique matrix remodeling machinery. It is not surprising that, alteration in the ECM leads to disc degeneration and in some cases are the consequence of degenerative processes. Ultimately, ECM disruption leads to loss of mechanical properties and increase disc stiffness [9,44,59]. Noteworthy, disc degeneration can be divided in different subtypes such as: disc fibrosis, disc calcification and disc herniations [57]. Interestingly, the ECM presents a unique signature in each of these degenerative phenotypes. While fibrotic degeneration seems to be promoted by secretion of collagenous matrix by hypertrophic-like cells, with increased PGs catabolism; intervertebral disc mineralization is more correlated with microenvironment changes induced by high levels of calcium, phosphate, cell death and TNAP activity. On the other hand, disc herniation, appears more depend on altered mechanical stresses and accelerated AF degeneration over NP, with loss of ECM structural molecules, such as COL1 or COL2. Another important factor relevant to disc degeneration is the role of aging and cellular senescence, having a considerable association with senescence associated secretory phenotype and matrix composition. In summary, understanding homeostatic mechanisms in the disc that govern ECM composition and quality provides insights into multifactorial pathogenesis of intervertebral disc degeneration.

## Declaration of competing interest

The authors declare that they have no known competing financial interests or personal relationships that could have appeared to influence the work reported in this paper.

## References

[1] Mokdad A.H., Ballestros K., Echko M., Glenn S., Olsen H.E., Mullany E., Lee A., Khan A.R., Ahmadi A., Ferrari A.J., Kasaeian A., Werdecker A., Carter A., Zipkin B., Sartorius B., Serdar B., Sykes B.L., Troeger C., Fitzmaurice C., Rehm C.D., Santomauro D., Kim D., Colombara D., Schwebel D.C., Tsoi D., Kolte D., Nsoesie E., Nichols E., Oren E., Charlson F.J., Patton G.C., Roth G.A., Hosgood H.D., Whiteford H.A., Kyu H., Erskine H.E., Huang H., Martopullo I., Singh J.A., Nachega J.B., Sanabria J.R., Abbas K., Ong K., Tabb K., Krohn K.J., Cornaby L., Degenhardt L., Moses M., Farvid M., Griswold M., Criqui M., Bell M., Nguyen M., Wallin M., Mirarefin M., Qorbani M., Younis M., Fullman N., Liu P., Briant P., Gona P., Havmoller R., Leung R., Kimokoti R., Bazargan-Hejazi S., Hay S.I., Yadgir S., Biryukov S., Vollset S.E., Alam T., Frank T., Farid T., Miller T., Vos T., Bärnighausen T., Gebrehiwot T.T., Yano Y., Al-Aly Z., Mehari A., Handal A., Kandel A., Anderson B., Biroscak B., Mozaffarian D., Dorsey E.R., Ding E.L., Park E.-K., Wagner G., Hu G., Chen H., Sunshine J.E., Khubchandani J., Leasher J., Leung J., Salomon J., Unutzer J., Cahill L., Cooper L., Horino M., Brauer M., Breitborde N., Hotez P., Topor-Madry R., Soneji S., Stranges S., James S., Amrock S., Jayaraman S., Patel T., Akinyemiju T., Skirbekk V., Kinfu Y., Bhutta Z., Jonas J.B., Murray C.J.L., U.S.B.o.D. Collaborators (2018). The state of US health, 1990–2016: burden of diseases, injuries, and risk factors among US states. JAMA.

[2] Iozzo R.V., Gubbiotti M.A. (2018). Extracellular matrix: the driving force of mammalian diseases. Matrix Biol..

[3] Shapiro I.M., Vresilovic E.J., Risbud M.V. (2012). Is the spinal motion segment a diarthrodial polyaxial joint: what a nice nucleus like you doing in a joint like this?. Bone.

[4] Johnson Z.I., Shapiro I.M., Risbud M.V. (2014). Extracellular osmolarity regulates matrix homeostasis in the intervertebral disc and articular cartilage: evolving role of TonEBP. Matrix Biol..

[5] Pan H., Strickland A., Madhu V., Johnson Z.I., Chand S.N., Brody J.R., Fertala A., Zheng Z., Shapiro I.M., Risbud M.V. (2019). RNA binding protein HuR regulates extracellular matrix gene expression and pH homeostasis independent of controlling HIF-1alpha signaling in nucleus pulposus cells. Matrix Biol..

[6] Choi H., Merceron C., Mangiavini L., Seifert E.L., Schipani E., Shapiro I.M., Risbud M.V. (2016). Hypoxia promotes noncanonical autophagy in nucleus pulposus cells independent of MTOR and HIF1A signaling. Autophagy.

[7] Silagi E.S., Batista P., Shapiro I.M., Risbud M.V. (2018). Expression of carbonic anhydrase III, a nucleus pulposus phenotypic marker, is hypoxia-responsive and confers protection from oxidative stress-induced cell death. Sci. Rep..

[8] Schoepflin Z.R., Silagi E.S., Shapiro I.M., Risbud M.V. (2017). PHD3 is a transcriptional coactivator of HIF-1α in nucleus pulposus cells independent of the PKM2-JMJD5 axis. FASEB J..

[9] Choi H., Tessier S., Silagi E.S., Kyada R., Yousefi F., Pleshko N., Shapiro I.M., Risbud M.V. (2018). A novel mouse model of intervertebral disc degeneration shows altered cell fate and matrix homeostasis. Matrix Biol..

[10] Zhang Y., Xiong C., Kudelko M., Li Y., Wang C., Wong Y.L., Tam V., Rai M.F., Cheverud J., Lawson H.A., Sandell L., Chan W.C.W., Cheah K.S.E., Sham P.C., Chan D. (2018). Early onset of disc degeneration in SM/J mice is associated with changes in ion transport systems and fibrotic events. Matrix Biol..

[11] Tessier S., Tran V.A., Ottone O.K., Novais E.J., Doolittle A., DiMuzio M.J., Shapiro I.M., Risbud M.V. (2019). TonEBP-deficiency accelerates intervertebral disc degeneration underscored by matrix remodeling, cytoskeletal rearrangements, and changes in proinflammatory gene expression. Matrix Biol..

[12] Mayer J.E., Iatridis J.C., Chan D., Qureshi S.A., Gottesman O., Hecht A.C. (2013). Genetic polymorphisms associated with intervertebral disc degeneration. Spine J..

[13] Deng Y., Tan X.T., Wu Q., Wang X. (2017). Correlations between COL2A and aggrecan genetic polymorphisms and the risk and clinicopathological features of intervertebral disc degeneration in a Chinese Han population: a case-control study. Genet. Test. Mol. Biomarkers.

[14] Silagi E.S., Shapiro I.M., Risbud M.V. (2018). Glycosaminoglycan synthesis in the nucleus pulposus: dysregulation and the pathogenesis of disc degeneration. Matrix Biol..

[15] Sivan S.S., Wachtel E., Roughley P. (2014). Structure, function, aging and turnover of aggrecan in the intervertebral disc. Biochim. Biophys. Acta.

[16] Collin E.C., Carroll O., Kilcoyne M., Peroglio M., See E., Hendig D., Alini M., Grad S., Pandit A. (2017). Ageing affects chondroitin sulfates and their synthetic enzymes in the intervertebral disc. Signal Transduct. Target Ther..

[17] Wight T.N. (2017). Provisional matrix: a role for versican and hyaluronan. Matrix Biol..

[18] Binch A.L.A., Shapiro I.M., Risbud M.V. (2016). Syndecan-4 in intervertebral disc and cartilage: saint or synner?. Matrix Biol..

[19] Önnerfjord P., Khabut A., Reinholt F.P., Svensson O., Heinegård D. (2012). Quantitative proteomic analysis of eight cartilaginous tissues reveals characteristic differences as well as similarities between subgroups. J. Biol. Chem..

[20] Oegema T.R., Johnson S.L., Aguiar D.J., Ogilvie J.W. (2000). Fibronectin and its fragments increase with degeneration in the human intervertebral disc. Spine.

[21] Urano T., Narusawa K.i., Shiraki M., Sasaki N., Hosoi T., Ouchi Y., Nakamura T., Inoue S. (2011). Single-nucleotide polymorphism in the hyaluronan and proteoglycan link protein 1 (HAPLN1) gene is associated with spinal osteophyte formation and disc degeneration in Japanese women. Eur. Spine J..

[22] Kyriakides T.R., Zhu Y.H., Smith L.T., Bain S.D., Yang Z., Lin M.T., Danielson K.G., Iozzo R.V., LaMarca M., McKinney C.E., Ginns E.I., Bornstein P. (1998). Mice that lack thrombospondin 2 display connective tissue abnormalities that are associated with disordered collagen fibrillogenesis, an increased vascular density, and a bleeding diathesis. J. Cell Biol..

[23] Seki S., Kawaguchi Y., Chiba K., Mikami Y., Kizawa H., Oya T., Mio F., Mori M., Miyamoto Y., Masuda I., Tsunoda T., Kamata M., Kubo T., Toyama Y., Kimura T., Nakamura Y., Ikegawa S. (2005). A functional SNP in CILP, encoding cartilage intermediate layer protein, is associated with susceptibility to lumbar disc disease. Nat. Genet..

[24] Aszódi A., Chan D., Hunziker E., Bateman J.F., Fässler R. (1998). Collagen II is essential for the removal of the notochord and the formation of intervertebral discs. J. Cell Biol..

[25] Savontaus M., Metsäranta M., Vuorio E. (1997). Mutation in type II collagen gene disturbs spinal development and gene expression patterns in transgenic Del1 mice. Lab. Invest..

[26] Sahlman J., Pitkänen M.T., Prockop D.J., Arita M., Li S.W., Helminen H.J., Långsjö T.K., Puustjärvi K., Lammi M.J. (2004). A human COL2A1 gene with an Arg519Cys mutation causes osteochondrodysplasia in transgenic mice. Arthritis Rheum..

[27] Boyd L.M., Richardson W.J., Allen K.D., Flahiff C., Jing L., Li Y., Chen J., Setton L.A. (2008). Early-onset degeneration of the intervertebral disc and vertebral end plate in mice deficient in type IX collagen. Arthritis Rheum..

[28] Tegeder I., Lötsch J. (2009). Current evidence for a modulation of low back pain by human genetic variants. J. Cell. Mol. Med..

[29] Chen J., Jing L., Gilchrist C.L., Richardson W.J., Fitch R.D., Setton L.A. (2009). Expression of laminin isoforms, receptors, and binding proteins unique to nucleus pulposus cells of immature intervertebral disc. Connect. Tissue Res..

[30] Roberts S. (2002). Disc morphology in health and disease. Biochem. Soc. Trans..

[31] Eyre D.R., Muir H. (1976). Types I and II collagens in intervertebral disc. Interchanging radial distributions in annulus fibrosus. Biochem. J..

[32] Cloyd J.M., Elliott D.M. (2007). Elastin content correlates with human disc degeneration in the anulus fibrosus and nucleus pulposus. Spine.

[33] Yu J., Tirlapur U., Fairbank J., Handford P., Roberts S., Winlove C.P., Cui Z., Urban J. (2007). Microfibrils, elastin fibres and collagen fibres in the human intervertebral disc and bovine tail disc. J. Anat..

[34] Yu J., Fairbank J.C., Roberts S., Urban J.P. (2005). The elastic fiber network of the anulus fibrosus of the normal and scoliotic human intervertebral disc. Spine.

[35] Kim K.W., Lim T.H., Kim J.G., Jeong S.T., Masuda K., An H.S. (2003). The origin of chondrocytes in the nucleus pulposus and histologic findings associated with the transition of a notochordal nucleus pulposus to a fibrocartilaginous nucleus pulposus in intact rabbit intervertebral discs. Spine.

[36] Sandell L.J., Morris N., Robbins J.R., Goldring M.B. (1991). Alternatively spliced type II procollagen mRNAs define distinct populations of cells during vertebral development: differential expression of the amino-propeptide. J. Cell Biol..

[37] Zhu Y., McAlinden A., Sandell L.J. (2001). Type IIA procollagen in development of the human intervertebral disc: regulated expression of the NH(2)-propeptide by enzymic processing reveals a unique developmental pathway. Dev. Dyn..

[38] Melrose J., Ghosh P., Taylor T.K. (2001). A comparative analysis of the differential spatial and temporal distributions of the large (aggrecan, versican) and small (decorin, biglycan, fibromodulin) proteoglycans of the intervertebral disc. J. Anat..

[39] Alini M., Eisenstein S.M., Ito K., Little C., Kettler A.A., Masuda K., Melrose J., Ralphs J., Stokes I., Wilke H.J. (2008). Are animal models useful for studying human disc disorders/degeneration?. Eur. Spine J..

[40] Dong D.M., Yao M., Liu B., Sun C.Y., Jiang Y.Q., Wang Y.S. (2007). Association between the -1306C/T polymorphism of matrix metalloproteinase-2 gene and lumbar disc disease in Chinese young adults. Eur. Spine J..

[41] Takahashi M., Haro H., Wakabayashi Y., Kawa-uchi T., Komori H., Shinomiya K. (2001). The association of degeneration of the intervertebral disc with 5a/6a polymorphism in the promoter of the human matrix metalloproteinase-3 gene. J. Bone Joint Surg. (Br.).

[42] Le Maitre C.L., Freemont A.J., Hoyland J.A. (2006). Human disc degeneration is associated with increased MMP 7 expression. Biotech. Histochem..

[43] Patil P., Dong Q., Wang D., Chang J., Wiley C., Demaria M., Lee J., Kang J., Niedernhofer L.J., Robbins P.D., Sowa G., Campisi J., Zhou D., Vo N. (2019). Systemic clearance of p16(INK4a) -positive senescent cells mitigates age-associated intervertebral disc degeneration. Aging Cell.

[44] Pockert A.J., Richardson S.M., Le Maitre C.L., Lyon M., Deakin J.A., Buttle D.J., Freemont A.J., Hoyland J.A. (2009). Modified expression of the ADAMTS enzymes and tissue inhibitor of metalloproteinases 3 during human intervertebral disc degeneration. Arthritis Rheum..

[45] Akhatib B., Onnerfjord P., Gawri R., Ouellet J., Jarzem P., Heinegård D., Mort J., Roughley P., Haglund L. (2013). Chondroadherin fragmentation mediated by the protease HTRA1 distinguishes human intervertebral disc degeneration from normal aging. J. Biol. Chem..

[46] Johnson Z.I., Shapiro I.M., Risbud M.V. (2016). RNA sequencing reveals a role of TonEBP transcription factor in regulation of pro-inflammatory genes in response to hyperosmolarity in healthy nucleus pulposus cells: a homeostatic response?. J. Biol. Chem..

[47] Novais E.J., Diekman B.O., Shapiro I.M., Risbud M.V. (2019). p16(Ink4a) deletion in cells of the intervertebral disc affects their matrix homeostasis and senescence associated secretory phenotype without altering onset of senescence. Matrix Biol..

[48] Gorth D.J., Shapiro I.M., Risbud M.V. (2019). A new understanding of the role of IL-1 in age-related intervertebral disc degeneration in a murine model. J. Bone Miner. Res..

[49] Risbud M.V., Shapiro I.M. (2014). Role of cytokines in intervertebral disc degeneration: pain and disc content. Nat. Rev. Rheumatol..

[50] Ponnappan R.K., Markova D.Z., Antonio P.J.D., Murray H.B., Vaccaro A.R., Shapiro I.M., Anderson D.G., Albert T.J., Risbud M.V. (2011). An organ culture system to model early degenerative changes of the intervertebral disc. Arthritis Res. Ther..

[51] Choi H., Chaiyamongkol W., Doolittle A.C., Johnson Z.I., Gogate S.S., Schoepflin Z.R., Shapiro I.M., Risbud M.V. (2018). COX-2 expression mediated by calcium-TonEBP signaling axis under hyperosmotic conditions serves osmoprotective function in nucleus pulposus cells. J. Biol. Chem..

[52] Setton L.A., Chen J. (2006). Mechanobiology of the intervertebral disc and relevance to disc degeneration. J. Bone Joint Surg. Am..

[53] Fujita N., Hirose Y., Tran C.M., Chiba K., Miyamoto T., Toyama Y., Shapiro I.M., Risbud M.V. (2014). HIF-1-PHD2 axis controls expression of syndecan 4 in nucleus pulposus cells. FASEB J..

[54] Wang J., Markova D., Anderson D.G., Zheng Z., Shapiro I.M., Risbud M.V. (2011). TNF-α and IL-1β promote a disintegrin-like and metalloprotease with thrombospondin type I motif-5-mediated aggrecan degradation through syndecan-4 in intervertebral disc. J. Biol. Chem..

[55] Wang X., Wang H., Yang H., Li J., Cai Q., Shapiro I.M., Risbud M.V. (2014). Tumor necrosis factor-α- and interleukin-1β-dependent matrix metalloproteinase-3 expression in nucleus pulposus cells requires cooperative signaling via syndecan 4 and mitogen-activated protein kinase-NF-κB axis: implications in inflammatory disc disease. Am. J. Pathol..

[56] Vo N.V., Hartman R.A., Patil P.R., Risbud M.V., Kletsas D., Iatridis J.C., Hoyland J.A., Le Maitre C.L., Sowa G.A., Kang J.D. (2016). Molecular mechanisms of biological aging in intervertebral discs. J. Orthop. Res..

[57] Cheung K.M.C., Karppinen J., Chan D., Ho D.W.H., Song Y.-Q., Sham P., Cheah K.S.E., Leong J.C.Y., Luk K.D.K. (2009). Prevalence and pattern of lumbar magnetic resonance imaging changes in a population study of one thousand forty-three individuals. Spine.

[58] Eyre D.R., Muir H. (1977). Quantitative analysis of types I and II collagens in human intervertebral discs at various ages. Biochim. Biophys. Acta.

[59] Caldeira J., Santa C., Osório H., Molinos M., Manadas B., Gonçalves R., Barbosa M. (2017). Matrisome profiling during intervertebral disc development and ageing. Sci. Rep..

[60] Ngo K., Patil P., McGowan S.J., Niedernhofer L.J., Robbins P.D., Kang J., Sowa G., Vo N. (2017). Senescent intervertebral disc cells exhibit perturbed matrix homeostasis phenotype. Mech. Ageing Dev..

[61] Karamanos N.K., Theocharis A.D., Neill T., Iozzo R.V. (2019). Matrix modeling and remodeling: a biological interplay regulating tissue homeostasis and diseases. Matrix Biol..

[62] Tessier S., Doolittle A.C., Sao K., Rotty J.D., Bear J.E., Ulici V., Loeser R.F., Shapiro I.M., Diekman B.O., Risbud M.V. (2020). Arp2/3 inactivation causes intervertebral disc and cartilage degeneration with dysregulated TonEBP-mediated osmoadaptation. JCI Insight.

[63] Gorth D.J., Shapiro I.M., Risbud M.V. (2018). Transgenic mice overexpressing human TNF-alpha experience early onset spontaneous intervertebral disc herniation in the absence of overt degeneration. Cell Death Dis..

[64] Gorth D.J., Ottone O.K., Shapiro I.M., Risbud M.V. (2020). Differential effect of long-term systemic exposure of TNFα on health of the annulus fibrosus and nucleus pulposus of the intervertebral disc. J. Bone Miner. Res..

[65] Kanayama M., Togawa D., Takahashi C., Terai T., Hashimoto T. (2009). Cross-sectional magnetic resonance imaging study of lumbar disc degeneration in 200 healthy individuals. J. Neurosurg. Spine.

[66] Hristova G.I., Jarzem P., Ouellet J.A., Roughley P.J., Epure L.M., Antoniou J., Mwale F. (2011). Calcification in human intervertebral disc degeneration and scoliosis. J. Orthop. Res..

[67] Pfirrmann C.W., Metzdorf A., Zanetti M., Hodler J., Boos N. (2001). Magnetic resonance classification of lumbar intervertebral disc degeneration. Spine.

[68] Teraguchi M., Yoshimura N., Hashizume H., Muraki S., Yamada H., Minamide A., Oka H., Ishimoto Y., Nagata K., Kagotani R., Takiguchi N., Akune T., Kawaguchi H., Nakamura K., Yoshida M. (2014). Prevalence and distribution of intervertebral disc degeneration over the entire spine in a population-based cohort: the Wakayama spine study. Osteoarthritis Cartilage.

[69] Ohnishi T., Sudo H., Tsujimoto T., Iwasaki N. (2018). Age-related spontaneous lumbar intervertebral disc degeneration in a mouse model. J. Orthop. Res..

[70] Battié M.C., Videman T., Kaprio J., Gibbons L.E., Gill K., Manninen H., Saarela J., Peltonen L. (2009). The Twin Spine Study: contributions to a changing view of disc degeneration. Spine J..

[71] Park J., Gebhardt M., Golovchenko S., Perez-Branguli F., Hattori T., Hartmann C., Zhou X., de Crombrugghe B., Stock M., Schneider H., von der Mark K. (2015). Dual pathways to endochondral osteoblasts: a novel chondrocyte-derived osteoprogenitor cell identified in hypertrophic cartilage. Biol. Open.

[72] Yee A., Lam M.P., Tam V., Chan W.C., Chu I.K., Cheah K.S., Cheung K.M., Chan D. (2016). Fibrotic-like changes in degenerate human intervertebral discs revealed by quantitative proteomic analysis. Osteoarthritis Cartilage.

[73] Roberts S., Bains M.A., Kwan A., Menage J., Eisenstein S.M. (1998). Type X collagen in the human invertebral disc: an indication of repair or remodelling?. Histochem. J..

[74] O’Connell G.D., Vresilovic E.J., Elliott D.M. (2011). Human intervertebral disc internal strain in compression: the effect of disc region, loading position, and degeneration. J. Orthop. Res..

[75] Chanchairujira K., Chung C.B., Kim J.Y., Papakonstantinou O., Lee M.H., Clopton P., Resnick D. (2004). Intervertebral disk calcification of the spine in an elderly population: radiographic prevalence, location, and distribution and correlation with spinal degeneration. Radiology.

[76] Zhao Y.P., Tian Q.Y., Liu B., Cuellar J., Richbourgh B., Jia T.H., Liu C.J. (2015). Progranulin knockout accelerates intervertebral disc degeneration in aging mice. Sci. Rep..

[77] Warraich S., Bone D.B.J., Quinonez D., Ii H., Choi D.-S., Holdsworth D.W., Drangova M., Dixon S.J., Séguin C.A., Hammond J.R. (2013). Loss of equilibrative nucleoside transporter 1 in mice leads to progressive ectopic mineralization of spinal tissues resembling diffuse idiopathic skeletal hyperostosis in humans. J. Bone Miner. Res..

[78] O'Young J., Liao Y., Xiao Y., Jalkanen J., Lajoie G., Karttunen M., Goldberg H.A., Hunter G.K. (2011). Matrix Gla protein inhibits ectopic calcification by a direct interaction with hydroxyapatite crystals. J. Am. Chem. Soc..

[79] Clarke M.C.H., Littlewood T.D., Figg N., Maguire J.J., Davenport A.P., Goddard M., Bennett M.R. (2008). Chronic apoptosis of vascular smooth muscle cells accelerates atherosclerosis and promotes calcification and medial degeneration. Circ. Res..

[80] Novais E.J., Tran V.A., Miao J., Slaver K., Sinensky A., Dyment N.A., Addya S., Szeri F., Wetering K.V.D., Shapiro I.M., Risbud M.V. (2020). Comparison of inbred mouse strains shows diverse phenotypic outcomes of intervertebral disc aging. Aging Cell.

[81] Rai M.F., Schmidt E.J., Hashimoto S., Cheverud J.M., Sandell L.J. (2015). Genetic loci that regulate ectopic calcification in response to knee trauma in LG/J by SM/J advanced intercross mice. J. Orthop. Res..

[82] Bachmeier B.E., Nerlich A., Mittermaier N., Weiler C., Lumenta C., Wuertz K., Boos N. (2009). Matrix metalloproteinase expression levels suggest distinct enzyme roles during lumbar disc herniation and degeneration. Eur. Spine J..

[83] Higuchi M., Abe K., Kaneda K. (1983). Changes in the nucleus pulposus of the intervertebral disc in bipedal mice. A light and electron microscopic study. Clin. Orthop. Relat. Res..

[84] Miyamoto S., Yonenobu K., Ono K. (1991). Experimental cervical spondylosis in the mouse. Spine.

[85] Hu M.H., Yang K.C., Chen Y.J., Sun Y.H., Lin F.H., Yang S.H. (2018). Optimization of puncture injury to rat caudal disc for mimicking early degeneration of intervertebral disc. J. Orthop. Res..

[86] Iatridis J.C., Michalek A.J., Purmessur D., Korecki C.L. (2009). Localized intervertebral disc injury leads to organ level changes in structure, cellularity, and biosynthesis. Cell. Mol. Bioeng..

[87] Silva A.J., Ferreira J.R., Cunha C., Corte-Real J.V., Bessa-Gonçalves M., Barbosa M.A., Santos S.G., Gonçalves R.M. (2019). Macrophages down-regulate gene expression of intervertebral disc degenerative markers under a pro-inflammatory microenvironment. Front. Immunol..

